# Geometrical multiscale tortuosity of desert ant walking trajectories

**DOI:** 10.1242/jeb.247104

**Published:** 2024-05-24

**Authors:** Harald Wolf, Nina Baldy, Sarah Elisabeth Pfeffer, Kai Schneider

**Affiliations:** ^1^ Institute of Neurobiology, Ulm University, Albert-Einstein-Allee 11, 89081 Ulm, Germany; ^2^ Institut de Mathématiques de Marseille, Aix-Marseille Université, CNRS, 13331 Marseille Cedex 3, France

**Keywords:** *Cataglyphis*, Goal approach, Systematic search, Brownian motion, Geometrical multiscale statistics, Path tortuosity, Coarse-grained curvature

## Abstract

Desert ants stand out as some of the most intriguing insect navigators, having captured the attention of scientists for decades. This includes the structure of walking trajectories during goal approach and search behaviour for the nest and familiar feeding sites. In the present study, we analysed such trajectories with regard to changes in walking direction. The directional change of the ants was quantified, i.e. an angle θ between trajectory increments of a given arclength λ was computed. This was done for different length scales λ, according to our goal of analysing desert ant path characteristics with respect to length scale. First, varying λ through more than two orders of magnitude demonstrated Brownian motion characteristics typical of the random walk component of search behaviour. Unexpectedly, this random walk component was also present in – supposedly rather linear – approach trajectories. Second, there were small but notable deviations from a uniform angle distribution that is characteristic of random walks. This was true for specific search situations, mostly close to the (virtual) goal position. And third, experience with a feeder position resulted in straighter approaches and more focused searches, which was also true for nest searches, albeit to a lesser extent. Taken together, these results both verify and extend previous studies on desert ant path characteristics. Of particular interest are the ubiquitous Brownian motion signatures and specific deviations thereof close to the goal position, indicative of unexpectedly structured search behaviour.

## Teaser

**Summary:** Lagrangian geometrical multiscale analysis of *Cataglyphis* desert ant walking trajectories revealed hitherto unrecognised details of search paths, for instance, variation in path tortuosity depending on goal distance.

## INTRODUCTION

**Fig. 1.**
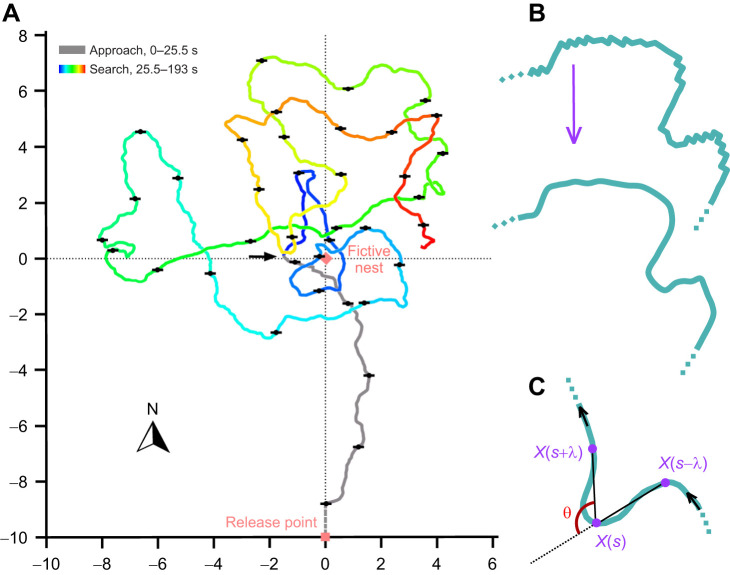
**Nest approach and nest search in desert ants, *Cataglyphis fortis*, and evaluation of walking trajectories.** (A) Walking trajectory of a forager ant homing in the test field. Black flags along the trajectory mark 5 s intervals, red square at the bottom the fictive feeder position (release point), red diamond in the centre the fictive nest position. The search path changes colour through the spectrum from blue at the start of the search to red at the end of the recording. Note the relatively straight approach path (grey, unusually bent in this sample recording, probably owing to brief western wind gusts during the approach) and the alternating spiralling of the search path; right turns, blue to turquoise and yellow to red; left turns, turquoise to yellow. N, compass north. (B) Sample trace segment indicating Bezier smoothing of digitised and thus square-cut trace recordings prior to further evaluation. (C) Sample trace segment indicating how a change in walking direction, angle θ, was determined for a (selected) trajectory increment arclength λ; δ*X*(*s*, λ)=*X*(*s*+λ)−*X*(*s*).

Desert ants, including the North African species *Cataglyphis fortis* studied here, are guided during their foraging excursions by a suite of orientation and navigation mechanisms. In barren desert biotopes, path integration is typically their primary navigation mechanism (Müller and Wehner, 1988; Ronacher, 2008; Wehner, 2020, 2009). Path integration allows the ants to return to the nest along an almost straight path (Fig. 1A, grey approach path) after lengthy meandering foraging trips, and it also serves the direct return to a plentiful food source encountered previously (Pfeffer et al., 2015; Bolek, 2012). These almost linear approaches to a goal, nest or feeder contrast with search behaviour that follows when the goal has not been encountered at the position indicated by the animal's path integrator (Fig. 1A, coloured search path). Search behaviour is characterised by a random walk superimposed on a search spiral centred on the assumed goal position (Müller and Wehner, 1994; Wehner and Srinivasan, 1981). Every so often, the direction of the search spiral reverses, and search spirals grow ever wider with increasing search time. During the search, the ant also occasionally returns close to the assumed goal position.

Goal approach and search behaviour have been analysed in much detail previously, desert ants included (above and Schultheiss et al., 2016; Bolek and Wolf, 2015). Typical parameters used to characterise and differentiate path trajectories include search density (time spent or distance travelled within a unit of searched area), path straightness (beeline distance between start and goal divided by actual path length) or directional fidelity (scatter of walking directions around the start – goal direction). However, further details of the walking trajectories have not received much attention. Here, we applied a novel measure to characterise walking trajectories, namely, path tortuosity, employing geometrical Lagrangian statistics (Burov et al., 2013). We determined tortuosity as the angular deviation θ between the start and end of a trajectory increment (correctly, its arclength; see below) of a particular length λ, averaged along the walking trajectory of interest. Using advanced Lagrangian statistics, we show how the multiscale character of the ant trajectories can be revealed by considering the distance lag dependence of directional change. This scale-dependent tortuosity yields insights into the complex multiscale dynamics of ant trajectories. The term tortuosity is used here synonymously to curvature, as used in previous work (Bos et al., 2015). It is defined as a measure for the deviation of a curve from a straight line. The term tortuosity, however, may better capture the winding character of ant search trajectories in everyday language.

The length λ of adjacent path increments may be chosen to analyse tortuosity across different length scales. A linear scaling of angle θ for small increments λ has been shown by Bos et al. (2015) to follow directly from the smoothness of the trajectories. They used second-order Taylor expansion, assuming that the velocity (here, speed of the ant) is constant for small increments. This approach has been fruitful in the time domain, where multiscale analyses of processes from molecular motion (Burov et al., 2013) to football games (Kadoch et al., 2017) have provided significant insights. In the present work, we replaced the time domain by the spatial domain and identify the arclength of trajectory increments with distance. For easier reading, we shall abbreviate the arclength of trajectory increments as trajectory or path increments.

In this study, we observed characteristics of the multi-scale structure of approach and search trajectories that have not been evident in the analysis methods used so far. Specifically, we analysed features of the ant trajectories demonstrated by multiscale analysis such as Brownian motion, the role of experience for trajectory characteristics, and apparent adjustment of search density according to experience and motivation, particularly close to the assumed goal position.

## MATERIALS AND METHODS

### Recording ant walking trajectories

Trajectories of the desert ant species *Cataglyphis fortis* (Forel, 1902; Wehner, 1983) were evaluated. They had been recorded in the context of a previous research project (Pfeffer et al., 2015, see their fig. 1). Details of the experimental procedures are provided in that publication.

In brief, an invisible feeding site was established at a distance of 10 m from the nest entrance. The sparse feeder contained five biscuit crumbs, the plentiful feeder many (>800). After picking up a food item, the ants started their home run and usually soon re-appeared to exploit the feeding site further. Ants visiting the feeder were marked with a colour code for individual identification. Each (thus marked) ant was used only once to record its approach and subsequent search path. That is, the number of evaluated trajectories equals the number of experimental ants (Table 1).

**Table 1.**
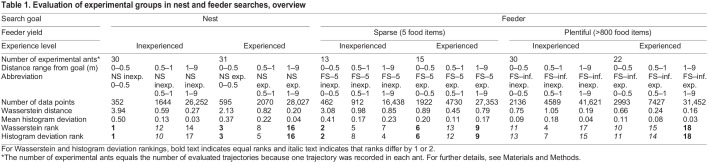
Evaluation of experimental groups in nest and feeder searches, overview

To record walking trajectories, 2×2 m grids were painted on the desert floor, their total dimensions 20×20 m. To record nest searches, we captured ants that had taken up a food item directly on the feeder and transferred them to a remote test field. Both the ants' homing runs with the food morsel to the fictive nest site and their subsequent nest search behaviour were documented. To record food site searches, the painted recording grid was centred on the feeding site, with the feeder removed in test situations (Wolf, 2008). Walking trajectories were traced on graph paper while following and observing an ant during its approach to and search for the feeder. Recording time was 2.5 min after an ant had started its search.

We considered an individual ant as inexperienced if its search behaviour was examined directly after the first visit to the feeder, that is, it did not have previous experience with the feeder location. This was true for both return to the nest and subsequent revisit of the feeding site. Experienced ants had visited the feeder and thus covered the nest–feeder route at least five times before they were tested, and usually more often (Wolf and Wehner, 2005; Wolf, 2008). We thus recorded approach and subsequent search trajectories for three different types of goals: the nest, a sparse feeding site and a plentiful feeding site. Each of these goal types was examined for inexperienced and experienced foragers, resulting in six experimental groups, with both approach and search examined. These groups and their abbreviations throughout the following text are listed in Table 1.

The recordings of goal approach and search behaviour on graph paper were digitised on a graphics tablet (Wacom Intuos 3, Wacom Europe GmbH, Krefeld, Germany). We evaluated separately the rather straight approach to the goal – nest or feeder – and the spiralling search behaviour. To separate approach from search trajectories, we identified the first conspicuous turning point in the recorded runs. This was defined as the point of the walking trajectory where an ant started to deviate from its current path direction by at least 30 deg and did not revert to the previous path direction for at least another 3 m (e.g. Merkle et al., 2006; Merkle and Wehner, 2010; arrow in Fig. 1A). The resolution of the recordings after digitisation, determined as the distance between neighbouring points of the digitised trajectory, was between 4 and 10 cm. It was thus commensurate with estimates of the recording accuracy on graph paper (p. 29 ff. in Bolek, 2012). All search trajectories were evaluated up to 12.80 m from the start, commensurate with the length of the shortest recorded search, to provide equal trajectory lengths for the 141 search path records. The corresponding approach path segments were often shorter because nest–feeder distance was 10 m.

### Choice of λ value for evaluation

**Fig. 2.**
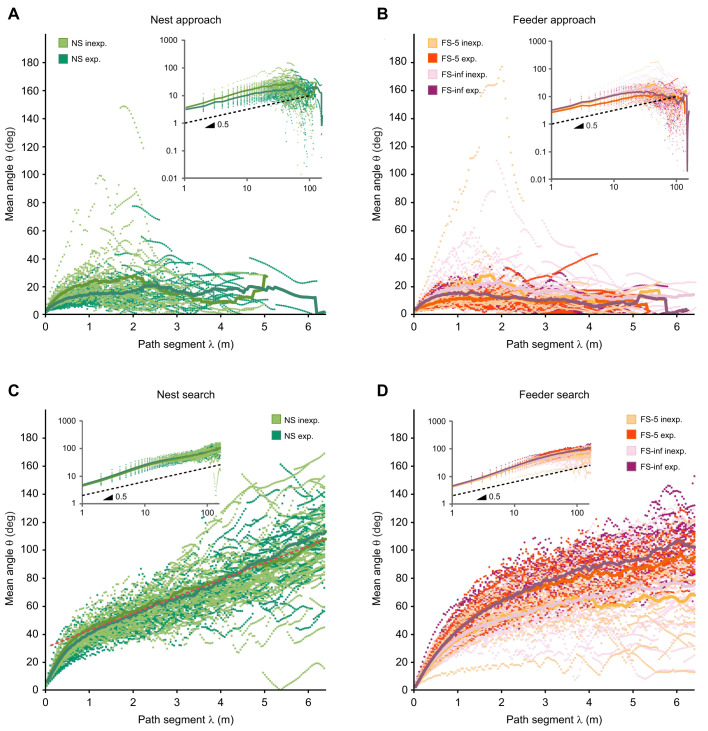
**Multiscale analysis of curvature angles (path tortuosity).** Mean changes in walking direction, angle θ (ordinate), are plotted against analysed trajectory increment arclengths, scale λ (abscissa). λ was varied from 0.04 to 6.40 m in 0.04 m increments. Small data points represent mean angles θ measured for a given walking trajectory at the given λ scale noted on the abscissa. For details, see Materials and Methods. (Grand) Mean angle curves of all evaluated trajectories (corresponding to ant numbers in Table 1) are drawn as solid lines. Note that θ=0 deg corresponds to straight paths, 90 deg to left/right turns, 180 deg to U-turns; trajectory arclength λ is a length scale, small λ indicating a short analysed distance (small scale) and large λ indicating a long distance (large scale). (A,C) Nest approaches and searches (shades of green); (B,D) food approaches and searches (shades of red/orange, sparse feeder, and violet, plentiful feeder). Insets show log–log plots with slopes of 0.5 indicated as dashed lines (indicative of Brownian movement characteristics). Linear course of nest search curves is indicated as red dashed line in C. Inset legends note experimental situations and colour codes: nest approaches (NA) and nest searches (NS) by inexperienced (inexp.) and experienced (exp.) forager ants; searches of inexperienced and experienced foragers for sparse (FS-5) and plentiful (FS-inf) feeding sites.

**Fig. 3.**
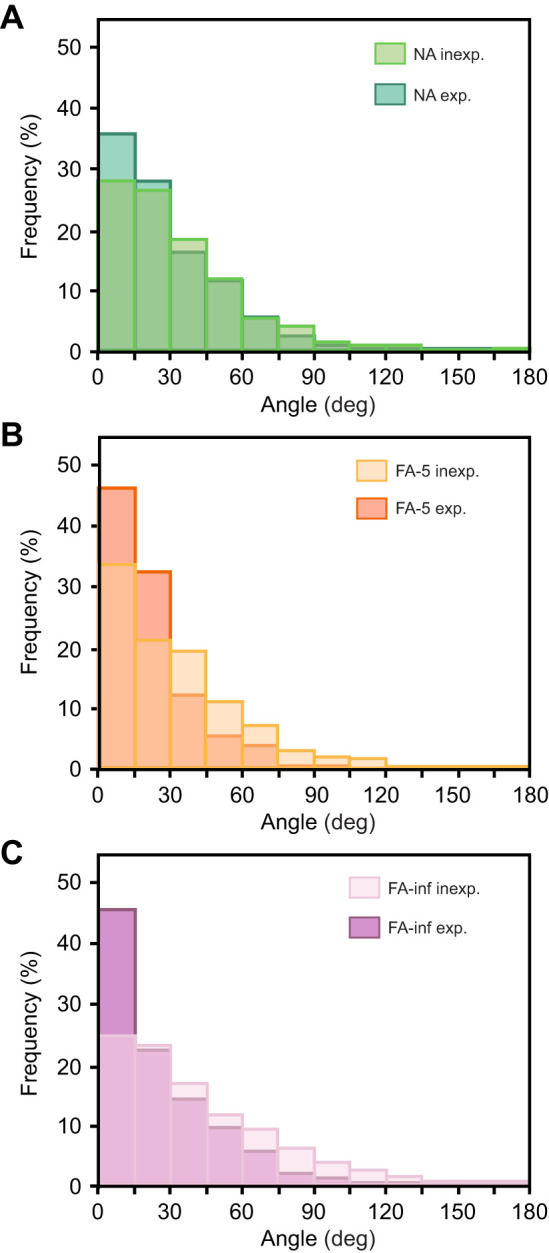
**Goal approaches by desert ants, *Cataglyphis fortis*.** Histograms of turning angle distributions for λ=50 cm. Goal approaches by inexperienced (light shades) and experienced (dark shades) foragers are plotted in the same diagram for comparison. (A) Nest approaches by foragers returning from a feeder (*N*=30 inexperienced/*N*=31 experienced ants). Trajectories were recorded after capturing the foragers at the feeder and displacement to the test field (situation depicted in Fig. 1A). (B) Forager approaches to a sparse feeder familiar from 1 (inexperienced, *N*=13) or >5 (experienced, *N*=15) previous visits. (C) Approaches to a plentiful feeder (>800 food items, *N*=30 inexperienced/*N*=22 experienced ants). Note the consistently narrower and higher distributions in experienced foragers.

**Fig. 4.**
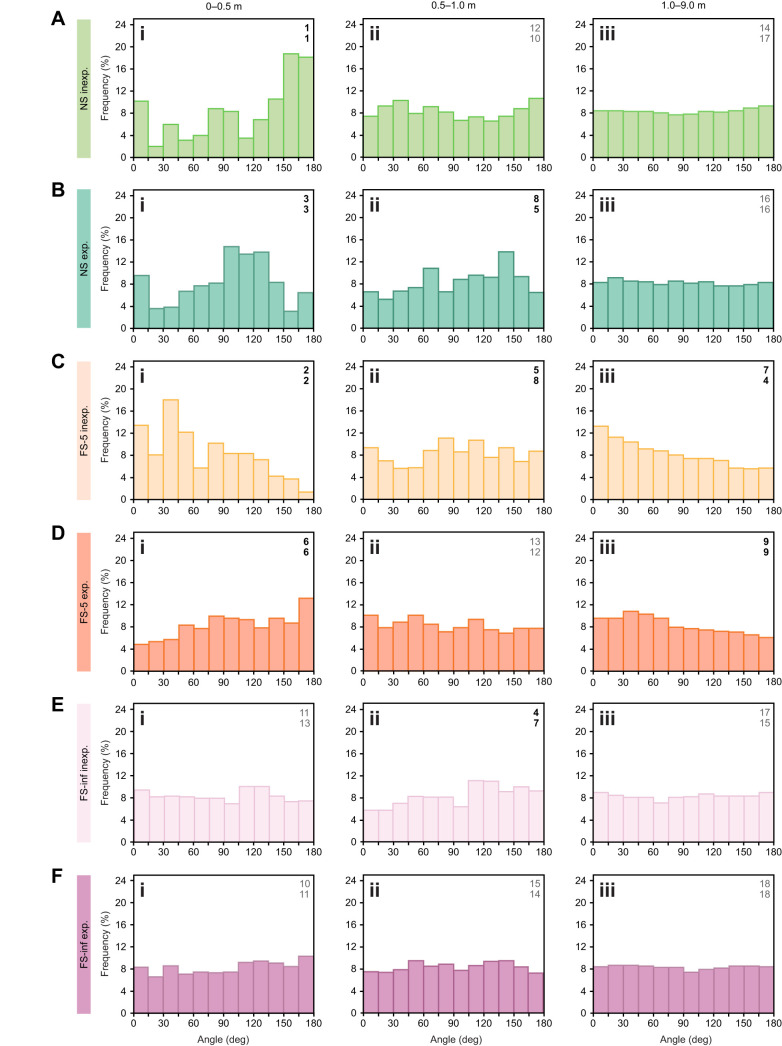
**Goal searches by desert ants, *Cataglyphis fortis*.** Histograms of turning angle distributions for λ=50 cm. Searches by inexperienced foragers are plotted in light shades, searches by experienced ants are plotted in dark shades. (A,B) Nest searches (green); (C,D) searches for sparse feeders (orange); (E,F) searches for plentiful feeders (violet). (i) Searches within 0.5 m distance from the (fictive) goal; (ii) searches between 0.5 and 1.0 m from the goal; (iii) searches between 1.0 and 9.0 m from the goal. Noted in the upper right corners of the diagrams are Wasserstein distance ranks (top numbers) and histogram deviation ranks (bottom numbers); ranks 1–9, black, ranks 10–18, grey. Numbers of experimental animals as in Fig. 3 and Table 1.

A scale value λ of 50 cm was selected to assess path tortuosity in desert ants' approach and search trajectories. This value was safely above human recording errors (estimated to be in the range of 10 to 20 cm; Bolek, 2012) and digitisation accuracy (above). At the same time, it was small enough to capture details of the ants' path characteristics that might be relevant for tortuosity evaluations. This includes the position of the 50 cm value beyond the strongest dependency of mean θ on λ range in our multiscale analysis (Fig. 2). Most importantly, a λ value of 50 cm proved appropriate for drawing up the histograms presented in Figs 3 and 4. In a preliminary set of evaluations, we constructed histograms for λ values ranging from 20 cm to 2 m. These demonstrated that 50 cm is appropriate to capture even small deviations from the expected uniform angle distribution, whereas with larger λ values such details tended to be lost.

### Multiscale angular statistics of trajectory curvature

The digitised ant traces were smoothed using Bézier curve-based interpolation (illustrated in Fig. 1B). Trajectories were divided into sub-trajectories of 40 digitisation points, which were then smoothed into Bézier sub-curves with the same number of points using De Casteljau's algorithm (Farin and Hansford, 2000). Smoothed ant trajectories were formed from the resulting sub-curves put end-to-end without discrepancy. Small loops and turns were preserved during this smoothing operation.

We carried out angular multiscale statistics to quantify the directional change of the walking trajectories. To measure directional changes, we computed the scale-dependent curvature angle (illustrated in Fig. 1C) using the formula introduced by Burov et al. (2013) and specified for Lagrangian trajectories in Bos et al. (2015), where we replaced time *t* by the arclength *s*. Given a set of recorded positions *X*(*s*) of a trajectory *X*:*s*→*X*(*s*), the Lagrangian spatial increment that yields vectors connecting positions separated by λ steps is defined as:
(1)

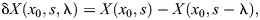
where *X*(*x*_0_, *s*) is the momentary position, passing through point *x*_0_ at trajectory increment *s*=*s*_0_ (Bos et al., 2015).

The angle between subsequent ant trajectory increments δ*X*(*x*_0_, *s*, λ) and δ*X*(*x*_0_, *s*+λ, λ) is described by its cosine, and θ emerges by applying the arccos:
(2)

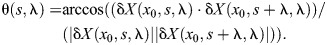
This is illustrated in Fig. 1C, and related to the curvature of the trajectory in the limit of short trajectory increments. For details, we refer to Bos et al. (2015). For short trajectory increments, we expect that θ(λ) tends to zero, which means that ants have the tendency of going straight. Exploiting how the angle varies in between short and long trajectory increment limits in different experimental settings is a major subject of this study.

Angles were plotted in the figures as absolute angles |θ|, that is, without distinction between right and left turns. For random motion and long analysed increments λ, we thus expect equi-partitioning of the angles corresponding to a mean value of 90 deg. First, there are no notable asymmetries in approach or search behaviour (e.g. Müller and Wehner, 1994; Wehner and Srinivasan, 1981), and second, the alternating spiralling of the search trajectories (Fig. 1A; see Introduction) should not bias our angle evaluations owing to balanced left and right turning angles.

We evaluated the recorded ant walking trajectories for λ scales ranging from 0.04 to 6.40 m in 0.04 m increments. 6.40 m is approximately half the length of the shortest recorded trajectory, thus setting the upper limit of 12.80 m evaluation length for all recordings. This resulted in 160 evaluations for each of the 141 recorded trajectories (total number of experimental ants in Table 1). Depending on the λ scale, between 319 (λ=0.04 m) and 1 (λ=6.40 m) angle measurements were performed for each trajectory (see Fig. 1C). The mean of all angle values for a given λ–trajectory combination was calculated, yielding one data point in Fig. 2. This data point represents mean path tortuosity (mean θ angle; ordinates in Fig. 2) for the examined length scale (trajectory increment length λ; abscissae in Fig. 2) in the given trajectory. Note that data points are arranged as dashed lines in outlier curves in Fig. 2, representing evaluations of a particular trajectory at successive λ values. These dashed lines illustrate the small but continuous changes in path tortuosity with incremental changes in the analysed length scale λ. Calculating the mean for all trajectories at a given λ illustrates the general scale dependency of path tortuosity (solid lines in Fig. 2).

### Data analysis and statistics

Statistical evaluation and testing [chi squared, goodness-of-fit, two-sample comparison and Wasserstein distance (also termed Vaserstein distance, depending on transcription)] were computed using their implementation in the Python package scipy.stats (e.g. Virtanen et al., 2020). We assessed the slopes of mean data curves (Fig. 2) with a Levenberg Marquardt algorithm (e.g. Transtrum and Sethna, 2012 preprint). Possible differences between approach trajectory sets from inexperienced and experienced ants (Fig. 3) were assessed with Epps–Singleton and Kolmogorov–Smirnov two-sample tests, the principle of which is to challenge the null hypothesis that two samples follow the same underlying probability distribution.

Regarding the data in Fig. 4, we wanted to assess potential deviations from the uniform distribution characteristic of random walk search behaviour. Classical statistical tests such as the chi-squared test appeared too dependent on histogram representation, in particular, numbers and widths of histogram bins, to provide a robust outcome suitable for further interpretation. Wasserstein distance is commonly used to compute distances between probability density functions (e.g. Panaretos and Zemel, 2019), here estimated using histograms. We thus used Wasserstein distances to assess potential non-uniform distributions of the data sets presented in Fig. 4 in histogram form. This was done by comparing those data sets with a uniform sample distribution (Vaserstein, 1969).

To further illustrate deviations from a uniform distribution, we calculated the mean of all data points used to construct a given histogram, and we took this mean as a reference for uniformity. Individual differences between mean and histogram bin values were summed across all 12 bins as absolute values. Division by the total number of data points in the histogram normalised the data point deviations across the different histograms. It is thus a measure of non-uniformity, although sensitive to bin height scatter. That scatter should be larger with smaller number of data points, that is, in the smaller distance ranges, particularly 0–0.5 m from the goal (see Results, section path tortuosity, search behaviour). We term this measure mean histogram deviation for the purpose of the present study.

Calculating Wasserstein distances and mean histogram deviations for the diagrams in Fig. 4 yielded rather similar results. That is, the three largest Wasserstein differences and mean histogram deviations occurred in the same data sets, as did several of the smaller differences. The actual numbers of Wasserstein distances and mean histogram deviations are not comparable, of course (Table 1). Therefore, we ranked the Wasserstein and histogram deviations, 1 to 18 according to the 18 histograms presented in Fig. 4. Ranks are provided in each of the diagrams in Fig. 4 and in Table 1. We did not consider ranks lower than 9 for further scrutiny, that is, Wasserstein distances <0.79 and histogram deviations <0.17. Visible deviations in Fig. 4 actually agree rather well with the ranking results, down to about rank 7.

## RESULTS

### Multiscale analysis

Analysis of movement trajectories across several orders of magnitude explores the characteristics of the respective movement and may indicate underlying complex mechanisms (Burov et al., 2013). This has been applied successfully to movements ranging from that of gas molecules to that of soccer players in the pitch (Kadoch et al., 2017). In the current case of goal approach and search trajectories in desert ants, analysis was limited to slightly more than two orders of magnitude. The lower limit of 4 cm was set by digitisation accuracy of the original graph paper records of approximately 4 cm. The upper limit was set by the length of the shortest analysed search trajectory, which had to accommodate two trajectory increments at least (see Materials and Methods; Fig. 1C). In Fig. 2, thus, the length scale λ of analysed trajectory increments on the abscissa ranges from 0.04 to 6.40 m. Angle values θ on the ordinate give the change in walking direction between the beginning and end of trajectory increments λ. Mean θ values are shown, calculated from all (absolute) angle values obtained in a particular walking trajectory for the analysed arclength λ.

Fig. 2A and B compare data for nest and feeder approaches. When approaching a goal, *C. fortis* ants take rather direct paths unless there are obstacles or notable wind that compromise a straight course (Wolf, 2008) (Fig. 1A). In keeping with this path alignment towards the goal, mean θ values in Fig. 2A,B stay mostly below 20 deg even at the largest λ values. At the smallest λ values, θ angles are close to 0 deg because changes in walking direction tend towards 0 deg with the shortening of analysed trajectory arclengths λ (see Materials and Methods). Beyond λ values of about 2.5 m, the curves bend downward, sometimes to below 10 deg. This behaviour is apparently due to the fact that across larger walking distances, maintenance of the direction towards the goal takes precedence over navigating smaller obstacles on the desert surface. The main and intriguing similarity between approach towards and search for a goal is revealed in plots with logarithmic scales on both axes, abscissa and ordinate (log–log plots) (inset graphs Fig. 2). The slopes of both nest and food approach curves are close to 0.5 across most of the λ range, a value indicative of Brownian motion behaviour (as opposed to ballistic behaviour characterised by a slope of 1.0; Bos et al., 2015).

Fig. 2C and D compare the results for nest and feeder search behaviour, with λ again ranging from 0.04 to 6.40 m. Mean θ values cover the range of angles from 0 to 170 deg. Towards the highest λ values, mean θ values approach 100 deg for food searches by experienced ants (Fig. 2D), and 110 deg for nest searches (Fig. 2C). This is notable because mean curvature angles of random walks are expected to evolve towards 90 deg with increasing λ, indicating equipartitioning of the angles. After all, animals having walked far enough to cover the whole angle range should produce mean angles representing the mean of all values between 0 and |180 deg|. The rise of the mean θ curves to 100 deg or above thus appears intriguing. It demonstrates a consistent tendency of the search trajectories – based on random walks – to bend along larger walking distances, which reflects the spiralling character of search trajectories. It further illustrates a more pronounced spiralling character of experienced feeder searches compared with inexperienced ones. That is, experienced ants appear to be more certain about feeder position, the centre of the search spiral, than their inexperienced nestmates. Mean θ curves of inexperienced ants searching for a feeder actually run consistently below the curves of experienced foragers and do not reach even 80 deg within the λ range examined. Apparently, they do not perform complete spirals, on average, but rather search the potential food source area more superficially and briefly.

Nest searches appear to exhibit even more pronounced spiralling than experienced food searches, indicated by the some 10 to 20 deg larger mean θ angle at the largest λ scale. At the same time, consistent differences in the mean θ curves between inexperienced and experienced foragers are absent in nest searches. Actually, both groups exhibit almost identical mean curvature angles, indicative of the absence of learning effects (but see Pfeffer et al., 2015 and Fig. 3C below). Also intriguing is the almost linear course of the mean θ curves beyond λ values of approximately 1.2 m. There is no indication of θ saturation up to the largest λ values examined, resulting in the rise of mean θ values to approximately 110 deg, as noted above. This continuous linear rise of mean θ angles with increasing sampling distance λ suggests constant spiralling behaviour throughout the recorded search distance. The slope of the linear rise is approximately 12 deg m^−1^, which may be interpreted as the average bend of the search spirals without the superimposed random walk.

Angles larger than 90 deg may also be regarded as a tendency for U-turns. There indeed occur rather sharp turns in the recorded search trajectories, often roughly reversing walking direction (compare Fig. 1A). When considering the search patterns on the whole, though, those turns would appear as elements of the ‘search spiral superimposed by random walk’ strategy rather than distinct U-turns, with more or less straight path segments before and after.

The log–log plots in Fig. 2 (inset graphs) exhibit almost linear courses along the first order of magnitude and up to another half order beyond. Their slopes are close to 0.5, as reported above. This is distinctive of power law behaviour and self-similarity. It means that there is no characteristic scale of search behaviour exhibited by the ants, again a hallmark of random search. Along the second order of magnitude, there are small downward bends of the curves and their slopes decline to just below 0.5.

It is remarkable that for both behavioural situations, approach and search trajectories, log–log plots are so similar along most of the graphs' courses, despite large differences in the lin–lin plots. This highlights not only the Brownian motion characteristics but also the illustrative power of the two types of graphic display. There are also distinct differences, most notably: (i) relatively small mean θ angles in goal approach compared with mean θ values well above 90 deg towards larger λ values in search behaviour, particularly nest searches; and (ii) experienced feeding site searchers exhibiting distinctly larger mean θ values than inexperienced foragers.

### Path tortuosity

#### Approach trajectories

According to the rather straight paths taken by goal-bound ant foragers, turning angles should be small, unless the walking terrain affords detours around (smaller) obstacles. Probability density functions in Fig. 3 plot the frequency of turning angles performed during goal approach. As to be expected, they peak around 0 deg and level off towards larger angles. That is, relatively straight path segments were most common in all analysed approach trajectories, independent of the experimental situation. However, there were clear and statistically significant differences (*P*<0.01; Epps–Singleton and Kolmogorov–Smirnov tests) between the angular distributions of inexperienced and experienced foragers. The experienced ants showed consistently narrower distributions, that is, less tortuous approach trajectories. This was particularly evident for the feeder searches (Fig. 3B,C), although the nest searches also exhibited statistically significant differences (Fig. 3A).

#### Search behaviour

Desert ant search behaviour exhibits a density peak at the search centre, the goal position assumed by the navigators, and it tapers out with increasing distance from the goal. This density profile is produced by the ants passing by the centre more often with their search spiral (superimposed with a random walk) being centred on the goal and occasional returns close to the (fictive) goal position (see Introduction; Müller and Wehner, 1994; Wehner and Srinivasan, 1981). Moreover, the search spirals grow wider with increasing search time. We wanted to examine whether path characteristics such as tortuosity also play a role. We thus evaluated the angular characteristics of search paths at different distances from the goal and in different behavioural situations. That is, we compared nest searches (Fig. 4A,B; top two diagram rows) and searches for sparse and plentiful feeding sites (Fig. 4C,D and E,F; middle and bottom two diagrams rows, respectively) in both inexperienced and experienced ants (Fig. 4A,C,E and B,D,F, respectively).

In an initial set of evaluations, we examined a larger set of distance ranges to identify possible deviations (e.g. Fig. 4Ciii) from the uniform angle distribution characteristic for random search (e.g. Fig. 4Eiii,Fiii). We examined the distance ranges of 0–0.5, 0–1, 0.5–1, 1–2, 1–9, 2–4, 2–8 and 4–8 m from the goal, and the complete recorded distance range, 0–9 m. Only in a few experimental situations did we observe notable deviations from a uniform angle distribution. This was mostly close to the search centre, namely, 0–0.5 m from the goal, and at larger distances, in the 1–9 m range. We thus chose for the final presentation three different distance ranges from the goal that captured all our notable observations while covering the whole search range: 0–0.5, 0.5–1 and 1–9 m (Fig. 4).

In the 0–0.5 m range from the goal (Fig. 4Ai–Fi, left column), the nest searches in particular exhibited discernible deviations from a uniform data distribution (Fig. 4Ai,Bi). Larger turning angles were recorded more frequently than expected from a uniform random walk distribution, and smaller turning angles occurred less frequently, which implies more tortuous and thus more focused search behaviour near the goal. This implication is supported by the largest Wasserstein distances from uniformity and the largest mean histogram deviations (see Materials and Methods for nest searches by inexperienced foragers; Fig. 4Ai). Both measures yielded ranks of 1 (Table 1; see Materials and Methods), and ranks of 3 were achieved by experienced ants searching for the nest. This was true despite the relatively large scatter of the histogram bins owing to the small number of data points. A total of 352 and 595 angle measurements were obtained in 30 inexperienced and 31 experienced ants, respectively, amounting to an average 8.2 m walking distance or 16.4 angle measurements per animal (Table 1). In the 0.5–1 m range from the goal, by comparison, an average 64 angle measurements were obtained per animal, and 936 measurements in the 1–9 m range. These larger numbers of data points notably reduced the scatter between histogram bins (compare diagram rows in Fig. 4Ai–iii and Bi–iii). Similar relationships were true for the other experimental groups presented in Fig. 4, with mostly larger data point numbers, up to 41,621 for searches of inexperienced foragers for a plentiful feeding site in the 1–9 m range (Table 1).

Notable deviations from uniformity were also observed for the searches of both experienced and inexperienced foragers for poorly supplied feeders (Fig. 4Ci,Di). Food site searches by inexperienced foragers achieved rank 2, and experienced foragers rank 6 in both Wasserstein distance and mean histogram deviation measures. The food searches by inexperienced ants showed an over-representation of small turning angles, contrasting with the food searches by experienced ants and with the nest searches presented above. Over-representation of small turning angles indicates relatively straighter walking trajectories and thus less focused food searches by ants not very familiar with or not much interested in the sparse feeding site.

In the 0.5–1 m range (Fig. 4Aii–Fii, middle column), there were much smaller though still discernible deviations from a uniform angle distribution. These minor deviations are visible in Fig. 4Bii for the nest searches by experienced ants, in Fig. 4Cii for searches by inexperienced ants for a sparse feeder, and in Fig. 4Eii for searches by inexperienced ants for a plentiful feeder. The clearly smaller deviations from uniform distributions are reflected by accordingly lower ranks, that is, by smaller Wasserstein distances and mean histogram deviations. Wasserstein distance and histogram deviation ranks were, respectively, 8 and 5, 5 and 8, and 4 and 7 (Table 1). In all three histograms, higher turning angles are slightly over-represented. In the case of the nest searches, this agrees with the situation in the 0–0.5 m range. In the searches for the sparse feeder, this is opposite to the 0–0.5 m range; and in the searches for the plentiful feeder, there is a rather uniform distribution in the 0–0.5 m range.

In the 1–9 m range (Fig. 4Aiii–Fiii, right column), far from the fictive goal, there are two histograms that exhibit discernible deviations from a uniform distribution. These are the inexperienced (Fig. 4Ciii) and the experienced (Fig. 4Diii) searches for a sparse feeder. The deviations are relatively small, and Wasserstein distance ranks and mean histogram deviation ranks are accordingly low (7 and 4, and 9 and 9, respectively; Table 1). Nonetheless, these observations are notable because the numbers of data points in the histograms are large, 16,438 and 27,353, respectively (Table 1). In both histograms, smaller turning angles are over-represented, that is, the ants took wider turns and followed straighter paths farther from the goal.

## DISCUSSION

### Multiscale analysis

Analysis of desert ants' search trajectories across a good two orders of magnitude of λ scale illustrated their well-known features (see Introduction) under new perspectives. The random walk component of the search trajectories is revealed by the log–log plots in Fig. 2C,D. The slope of the mean θ curves is close to 0.5, characteristic of Brownian motion behaviour and scale-independent self-similarity as a hallmark of random search. The spiralling component of search behaviour is represented by the mean θ angles (Fig. 2C,D) rising well above 90 deg, the average change in walking direction to be expected for longer walking distances λ in a random walk. The searches for familiar feeding sites depend in their expression on the degree of familiarity with feeder position and yield, and they may be rather transitory for less familiar and less attractive sparse feeders (Pfeffer et al., 2015). Nest searches, by contrast, maintain their basic structure over time periods limited only by the survival of the searching individual in the hot and barren desert environment. This agrees with another intriguing feature of the nest searches shown in Fig. 2C, the almost linear rise of the mean θ curves beyond λ values of approximately 1.2 m. These linear graph segments follow the equation:
(3)

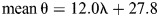
when pooling the data beyond λ=1.2 m for inexperienced and experienced nest searches (exhibiting similar mean θ curves; Fig. 2C). This linear rise apparently reflects the consistent spiralling feature of search trajectories, with a full 360 deg reached after approximately 30 m path length in the analysed initial 2.5 min of nest searches. Those 360 deg are, of course, a conceptual value obtained by summing all spiral fragments discernible in the search patterns.

Contrasting with nest searches, mean θ curves of feeder searches (Fig. 2D) follow power law functions (Fig. 2D, inset) in accord with expectation (see above). This flattening of mean θ curves, even to angles above 90 deg in experienced foragers, indicates that the spiralling component of feeder searches abates with search distance and becomes almost constant at the largest observed walking distances. This holds, although between λ values of about 1 and 5 m, mean θ angles of experienced feeder searches are actually slightly larger than those of nest searches. In this λ range, experienced feeder searches exhibit higher curvature angles and are thus more concentrated compared with nest searches, as reported previously in search density profiles (Pfeffer et al., 2015). Although this is true for experienced foragers, inexperienced foragers show consistently smaller mean θ angles, compared with all other search groups. Again, it has been reported previously that experience improves the search focus for familiar feeding sites, as does feeder yield (Pfeffer et al., 2015). This is in contrast to nest searches that improve little – though discernibly – with experience (except for dedicated learning walks of new foragers; Fleischmann et al., 2016; Fleischmann, 2019).

The approach trajectories, although basically straight advances towards the goal, exhibit intriguing similarities to the search trajectories with regard to their random walk characteristics (Fig. 2A,B, log–log inset graphs). Like in the nest searches, slopes of the mean θ curves are close to 0.5, indicative of random walks. Deviations from a straight course are probably introduced by environmental factors such as substrate structure and obstacles, particularly because ants are able to follow rather straight courses under laboratory conditions, on smooth and even walking substrates (Hangartner, 1967). Items that deflect homing ants from a straight course may well occur randomly and thus cause at least part of the random walk component superimposed on the straight goal-directed course. This aspect may be relevant also for search trajectories. In any case, search and approach trajectories share random walk properties at least on a roughly 4 to 40 cm scale, the distance range in focus when analysing desert ant walking path structure. The layout of search behaviour on a larger scale has been studied previously (see Introduction; Müller and Wehner, 1994; Wehner and Srinivasan, 1981).

### Path tortuosity

Despite the consistent random walk features, approach trajectories differ between inexperienced and experienced foragers. This holds for both outbound and homing paths, that is, nest (Fig. 3A) and feeder approaches, including differently stocked feeders (Fig. 3B,C). Inexperienced foragers follow more tortuous paths compared with ants well familiar with feeder position. Walking angle distributions are all centred on 0 deg, but are significantly broader for inexperienced as opposed to experienced foragers. This difference is least pronounced in nest approaches (Fig. 3A), again supporting the long-established notion that nest approach and search improve little with experience, compared with navigation with regard to more ephemeral objects such as food sources (Wolf, 2008; Pfeffer et al., 2015). Exceptions are learning walks at the beginning of an ant's foraging life (see above; Fleischmann et al., 2016). Nonetheless, there is improvement in the straightness of approach trajectories towards the nest with experience in the current analysis, presumably owing to the animals becoming familiar with the outbound and homebound paths and the major obstacles along these routes.

Angle distributions of search behaviour would be expected to be close to uniform distributions, according to the random walk characteristics discussed above. This proves true for most situations (Fig. 4), exemplified by the searches of experienced foragers for a well familiar and plentiful feeding site (Fig. 4F). However, there are notable exceptions with clear deviations from uniformity. This applies in particular close to the search centre, where paths are more tortuous in searches for the nest (Fig. 4Ai,Bi) and for sparsely equipped feeders by experienced ants (Fig. 4Di). The reverse pattern with straighter walking paths is evident in searches for a sparse feeder by inexperienced ants (Fig. 4Di). Far from the search centre, too, deviations occur in two histograms, with smaller curvature angles dominating over larger ones (Fig. 4Ciii,Diii).

More curved paths would appear to be useful by increasing search density close to the assumed goal position, here within 0.5 m from the goal (Fig. 4Ai,Bi,Di) or a little beyond (Fig. 4Bii,Eii). By contrast, the predominance of smaller turning angles close to the fictive goal is hard to interpret (Fig. 4Ci, possibly carrying over to Fig. 4Cii). It would appear that inexperienced ants returning to the neighbourhood of a sparse feeder do not expect to find another food morsel in in the area, but rather tend to follow their sector fidelity, as reported previously (Wehner et al., 1983, 2004). This interpretation certainly agrees with the rather transitory visits to less familiar and less plentiful feeding sites (Pfeffer et al., 2015). Far from the goal, predominance of smaller curvature angles will tend to reduce search density by widening search loops, which would appear to be useful for sparse feeding sites that may not be worth re-visiting.

### Conclusion and outlook

Studying desert ant goal approach and search trajectories with the novel geometrical multiscale tortuosity approach extended our understanding by a number of significant aspects. First, the random walk characteristics of search behaviour are intriguingly apparent in directed goal approach trajectories. This may be the result of random structural features of the walking substrate, an aspect perhaps also relevant in systematic searches, depending on locomotion substrate. Second, there are small but clear deviations from the uniform angle distribution characteristic of random walks. Apparently, search density is increased near the assumed goal position by more tortuous path characteristics in nest searches and searches for feeders with high familiarity and expected yield. Far from the goal, by contrast, search trajectories may be less tortuous, reducing search density. These aspects occur in addition to the well-studied search characteristics of desert ants such as search spiral with superimposed random walk. And third, experience with a given goal approach typically reduces directional scatter.

To address the development of search behaviour across larger search spirals, it is desirable to record much longer search trajectories in the future. It is further tempting to develop computer programs using machine learning and deep neural networks to simulate ant navigation behaviour and, in particular. goal approach and search behaviour. This would allow a deeper understanding of the multiscale characteristics of desert ant search behaviour, together with the recording of longer search trajectories.

All above agreements between our current multiscale analysis and previous accounts based on search density profiles prove multiscale analysis a valuable tool for trajectory assessment. This holds, in particular, in view of the new aspects revealed by the current method. This further illustrates the potential applicability and relevance to other organisms and research fields (e.g. Bartumeus et al., 2005; Benhamou and Collet, 2015).
